# Management of Femoral Periprosthetic Fractures: An Institutional Experience at a District General Hospital

**DOI:** 10.7759/cureus.74149

**Published:** 2024-11-21

**Authors:** Muhammad Muneeb Safdar, Jake Sumpton, Christopher Lodge

**Affiliations:** 1 Surgery, Musgrove Park Hospital, Taunton, GBR; 2 Trauma and Orthopaedics, Hull York Medical School, York, GBR; 3 Trauma and Orthopaedics, York Hospital, York, GBR

**Keywords:** femoral periprosthetic fractures, fixation, hip fractures, mortality, revision

## Abstract

Introduction

A total hip replacement is a common procedure performed by trauma and orthopaedic surgeons. Successful outcomes in arthroplasty surgery have significantly contributed to more hip replacement procedures being performed annually. This has also increased the incidence of femoral periprosthetic fractures, leading to more revision hip replacement procedures being performed.

Methods

This is a retrospective cohort study carried out at a district general hospital in the United Kingdom. Theatre records were reviewed from 2018 to 2022 to identify patients with a femoral periprosthetic fracture. Following the data collection, the patients were split into two groups. The first group analysed the patient outcomes through the type of surgical intervention they had, and the second group analysed the patients according to the timing of surgical intervention.

Results

There were 88 patients included in the study, out of which 49 had revision surgery and 39 had fixation for the femoral periprosthetic fracture. No statistically significant difference was observed in 30-day mortality and one-year mortality for the patients having revision or fixation surgery. Similarly, the results were not found to be significant in 30-day mortality and one-year mortality for the patients having surgery within 36 hours or after 36 hours of diagnosis.

Conclusion

Overall, the findings of this study are in keeping with the literature. Input from the specialist arthroplasty team is often required for the management of femoral periprosthetic fractures. There is no significant impact on mortality with a delay in surgical intervention for femoral periprosthetic fractures, unlike hip fractures. Medical optimisation and careful planning lead to better patient outcomes for this group of patients.

## Introduction

In trauma and orthopaedic surgery, a total hip replacement is one of the most common procedures performed by surgeons. As demonstrated by the National Joint Registry (NJR), there were a total of 105,798 hip procedures performed in 2022, compared to 89,634 hip procedures performed in 2021 [1]. It is also anticipated that the total number of hip arthroplasties being performed in the Organisation for Economic Cooperation and Development (OECD) countries will increase to approximately 2.8 million per year by 2050, compared to the 1.8 million that were performed in 2015 [2]. Due to the ageing population, coupled with successful outcomes, there has been an increase in the number of arthroplasty procedures being performed over the recent years [3]. An increase in the incidence of femoral periprosthetic fractures has been observed due to this [3, 4]. Out of all the total hip replacement revisions performed, it is estimated that about 15% are due to the periprosthetic fractures [3]. There was an increase in the number of revision total hip replacement procedures performed for femoral periprosthetic fractures from 922 in 2013 to 1240 in 2018 in the United Kingdom, excluding Scotland [4].

The patients, who sustain a femoral periprosthetic fracture after a total hip replacement, often have multiple co-morbidities that increase the risk of peri-operative complications. This can result in a delayed surgical intervention as patients are often medically optimised prior to surgery [5-8]. Delay in surgical intervention can also be due to the complex periprosthetic fracture pattern, requiring input from the specialist arthroplasty team as revision total hip replacements are often not performed by the general trauma and orthopaedic surgeons [7-10]. A delay in surgical intervention for femoral periprosthetic fractures is often due to a combination of medical optimisation, input from the specialist team and ensuring that all the equipment needed is available [7-10].

Several studies have demonstrated that a delay in surgical intervention for hip fractures could lead to an increase in the number of complications, including a higher mortality rate [11-14]. The implementation of the Best Practice Tariff (BPT) has played a significant role in the management of hip fractures; timing of surgical intervention is one of the criteria for BPT where it is recommended to perform the surgery within 36 hours of diagnosis [15]. Overall, a decline in the 30-day mortality rate was observed from 8.3% in 2009 to 6.1% in 2018 following the implementation of the BPT [5,16,17].

Unlike hip fractures, an increase in mortality has not been observed with a delay in surgical intervention for femoral periprosthetic fractures. A study by Boddice et al. demonstrated that the rate of mortality was not increased with a delay in surgical intervention for femoral periprosthetic fractures following a total hip replacement [5]. Another study by Singh et al. observed no statistically significant results for a higher mortality rate in periprosthetic fractures with a delay in surgical intervention of over 48 hours [18].

This study aims to add further evidence to the literature. We will be analysing whether a delay in the surgical intervention will lead to a higher mortality rate for femoral periprosthetic fractures. In addition to the mortality rate, we also aim to assess the post-operative complications associated with a delayed surgical intervention along with the factors that may predict post-operative complications.

## Materials and methods

This was a retrospective cohort study carried out at a district general hospital in the United Kingdom. To identify the patients for the study, the hospital’s theatre records were reviewed retrospectively from 2018 to 2022. The inclusion criteria consisted of the presence of a previous total hip replacement and a femoral periprosthetic fracture. The patients were excluded from the study if they were noted to have an implant fracture of the stem or a femoral periprosthetic fracture following a previous hip hemiarthroplasty, dynamic hip screw or a cephalomedullary nail. The patients were also excluded from the study if their periprosthetic fracture was managed conservatively.

Microsoft Excel (Microsoft® Corp., Redmond, WA, USA) was used to record the data on hospital computers. The data included consisted of the patient’s age, pre-operative haemoglobin value, total inpatient stay, post-operative stay after surgery, gender, date of surgical intervention, timing to surgical intervention, type of surgical intervention, post-operative complications, past medical history, American Society of Anaesthesiologists (ASA) grade, 30-day mortality and one-year mortality. The past medical history was used to calculate the Charlson Comorbidity Index (CCI) for the patients. The timing of X-rays was used as the time of diagnosis and the time for knife to skin was used as the time at which the surgical intervention was carried out.

Following the data collection, the patients were divided into two groups. The first group compared the patient outcomes according to the type of surgical intervention that was either a revision total hip replacement or fixation. The second group compared the patient outcomes according to the timing of surgical intervention where the patients having surgery within 36 hours were compared with the patients having surgery after 36 hours of diagnosis. The time of 36 hours was selected for this study as it is also recommended by BPT for hip fractures [13]. Our aim was to assess whether femoral periprosthetic fractures would have similar outcomes to hip fractures if they had a delay in surgical intervention.

The data was analysed using Statistical Package for the Social Sciences (SPSS) version 29 (IBM Corp., Armonk, NY, USA). Normality of the data was assessed through the Shapiro-Wilk test. The categorical data was assessed using Fisher’s exact test. The Mann-Whitney U test was used for data that was nonparametric. Regression tests were used to assess any associations for 30-day mortality, one-year mortality, post-operative complications, inpatient stay and post-operative stay.

## Results

Following the search of the theatre records, a total of 88 patients were identified that met the inclusion criteria of the study. The patient demographic data is demonstrated in Table 1. Out of the 88 patients, 49 (55.68%) had revision surgery and 39 (44.32%) had fixation for their femoral periprosthetic fracture. The mean age of the patient across both groups was 79.82 years. There were 41 males (46.59%) and 47 females (53.40%) in the study. The mean CCI across both groups was 4.55 and the individual means according to the surgical intervention are described in Table 1. It was observed that 40 patients (45.45%) had an ASA grade of 3.

**Table 1:** Demographic data and patient characteristics. ^ Fisher’s exact test * Mann-Whitney U test

	Revision (n=49)	Fixation (n=39)	P-Value
Mean age (SD)	78.04 (9.98)	82.05 (9.56)	0.078 *
Male (%)	26 (53.06%)	15 (38.46%)	0.125 ^
Female (%)	23 (46.93%)	24 (61.53%)	
Mean CCI (SD)	4.63 (1.85)	4.44 (1.45)	0.793 *
ASA Grade			
1 (%)	4 (8.16%)	0 (0%)	0.079 ^
2 (%)	24 (48.98%)	13 (33.33%)	
3 (%)	18 (36.73%)	22 (56.41%)	
4 (%)	3 (6.12%)	4 (10.26%)	

The patient outcomes, according to the type of surgical intervention, are described in Table 2. Although the 30-day mortality was higher in the revision group having four patients (8.16%), it was not found to be statistically significant. The one-year mortality was noted to be higher in the fixation group with four patients (10.26%) and it was also not found to be statistically significant. The median length of inpatient hospital stay was observed to be higher in the revision group (14 days). The median length of post-operative stay was higher for the patient who had fixation (10 days). However, the results for the inpatient and post-operative stay were not found to be statistically significant across both groups. Overall, 15 patients (17.05%) had surgical intervention within 36 hours of diagnosis, out of which 10 (20.41%) had revision surgery and 5 (12.82%) had fixation for their femoral periprosthetic fracture.

**Table 2:** Patient outcomes according to the type of surgical intervention. ^ Fisher’s exact test * Mann-Whitney U test

	Revision (n=49)	Fixation (n=39)	P-value
30-day mortality (%)	4 (8.16%)	2 (5.13%)	0.453 ^
One-year mortality (%)	2 (4.08%)	4 (10.26%)	0.237 ^
Median length of hospital stay (SD)	14 (19.44)	13 (8.60)	0.444 *
Median length of post-operative stay in hospital (SD)	9.00 (18.91)	10.00 (7.49)	0.257 *
Post-operative complications (%)	27 (55.10%)	18 (46.15%)	0.268 ^
Surgical intervention within 36 hours (%)	10 (20.41%)	5 (12.82%)	0.258 ^

Table 3 shows the patient outcomes according to the timing of surgical intervention. The 30-day and one-year mortality were both higher in patients who had surgery after 36 hours in comparison to patients having surgery within 36 hours; the results were not statistically significant for 30-day mortality and one-year mortality across both groups, as demonstrated in Table 3. The median length of inpatient stay and post-operative stay was higher for the patients who had surgery after 36 hours of diagnosis; the results were not statistically significant across these two groups with the p-values for inpatient stay and post-operative stay being 0.362 and 0.677, respectively.

**Table 3:** Patient outcomes according to the timing of surgical intervention. ^ Fisher’s exact test * Mann-Whitney U test

	Surgery <36 hours (n=15)	Surgery >36 hours (n=73)	P-Value
30-day mortality (%)	1 (6.67%)	5 (6.85%)	0.730 ^
One-year mortality (%)	1 (6.67%)	5 (6.85%)	0.730 ^
Median length of hospital stay (SD)	10.00 (17.88)	13.00 (15.59)	0.362 *
Median length of post-operative stay (SD)	9.00 (17.78)	10.00 (14.79)	0.677 *
Post-operative complications (%)	12 (80%)	33 (45.21%)	0.014 ^

The post-operative complications are demonstrated according to the type of surgical intervention and the timing of surgical intervention in Table 2 and Table 3, respectively. The following post-operative complications were observed: hospital-acquired pneumonia, blood transfusion post-operatively, inotropic support for maintaining blood pressure, leaky wound, cardiac arrest, loosening of acetabular component, acute kidney injury, non-ST-elevation myocardial infarction, urinary retention, dislocation, infection and subsequent washout in theatre. There were more post-operative complications in the revision group than the fixation group; the revision group had 27 patients (55.1%) who experienced a post-operative complication compared to 18 patients (46.15%) having post-operative complications in the fixation group, as demonstrated in Table 2. However, the results were not statistically significant. In contrast, more post-operative complications were observed in the group of patients who had surgery within 36 hours of diagnosis where 12 patients (80%) had a post-operative complication. There were 33 patients (45.21%) who had surgery after 36 hours of diagnosis and experienced a post-operative complication; this was statistically significant with a p-value of 0.014.

The multiple regression is demonstrated in Table 4 where the selected dependent variables were 30-day mortality, one-year mortality, post-operative complications, inpatient stay and post-operative stay. For the 30-day mortality variable, there was no significant relationship between the independent variables. There was a correlation between one-year mortality and age as well as CCI with p-values of 0.037 and 0.042, respectively. Furthermore, a correlation was demonstrated between post-operative complications and having surgical intervention after 36 hours, as demonstrated in Table 4. Out of the patients who had surgical intervention after 36 hours, one patient had acute coronary syndrome and one patient suffered a cardiac arrest postoperatively. A correlation was also observed between the ASA grade and inpatient as well as post-operative stay.

**Table 4:** Multiple linear regression for patient outcomes.

	30-day mortality p-value	One-year mortality p-value	Post-operative complications p-value	Inpatient stay p-value	Post-operative stay p-value
Age	0.356	0.037	0.606	0.224	0.143
Pre-operative haemoglobin	0.236	0.917	0.557	0.093	0.06
ASA grade	0.086	0.152	0.134	0.021	0.029
CCI	0.345	0.042	0.474	0.729	0.755
Surgical intervention after 36 hours	0.966	0.787	0.005	0.76	0.291

## Discussion

The results of our study are in keeping with the literature. In this study, most of the patients had surgical intervention after 36 hours whereas only 15 patients (17.05%) had surgery within 36 hours of diagnosis. The delay in surgical intervention was noted to be higher in the fixation group where 34 patients (87.18%) had surgery after 36 hours of diagnosis.

Delay in surgical intervention could be due to several factors. As femoral periprosthetic fractures are likely to be sustained in the cohort of patients with many comorbidities, medical optimisation is often required prior to surgical intervention to improve patient outcomes during the perioperative period. The availability of a specialist surgeon and additional imaging, such as computed tomography (CT), may contribute to delays in surgical intervention. If a patient, with a femoral periprosthetic fracture, is admitted on the weekend with no arthroplasty surgeon availability, they are likely to have delayed surgical intervention of over 36 hours.

The results of this study did not demonstrate any statistically significant difference between 30-day and one-year mortality for patients having their surgery within 36 hours or after 36 hours of diagnosis. An overall mortality of 6.82% at one year was observed in our study. A large study conducted by Boddapati et al. in New York did not observe any significant difference in 30-day mortality for patients having early or delayed surgery [7]. Despite the 30-day mortality results being similar to our study, Boddapati et al. did not assess mortality beyond 30 days [7]. Furthermore, the timing for early or delayed surgery was 24 hours in this study as opposed to the timing of 36 hours, which was selected for our study; nevertheless, this study had a large sample size of 857 patients [7]. Similarly, the study by Boddice et al. also did not observe any significant mortality for patients who had delayed surgical intervention for femoral periprosthetic fractures [5]; it was only the short-term mortality at 90 days that was assessed and the patients were divided according to the surgical intervention timing of 72 hours [5]. Although 30-day mortality was noted to be higher in the systematic review conducted by Farrow et al., this was not the case for mortality at 12 months [6].

The negative patient outcomes of delayed surgery in patients with hip fractures have been assessed in detail by several studies [19,20]. Simunovic et al. demonstrated a lower mortality rate along with improved patient outcomes for early surgery in patients with hip fractures [21]. In addition to the BPT recommending surgery within 36 hours [15], the general recommendation is to carry out surgical intervention within 48 hours for patients presenting with a hip fracture [22,23]. Given that hip fractures and femoral periprosthetic fractures are generally sustained in elderly patients with several comorbidities, one would expect to observe a similar pattern of mortality with delayed surgical intervention. However, a higher mortality rate has not been observed with delayed surgery in patients with femoral periprosthetic fractures [5,7] and the impact of delayed surgery on long-term mortality remains uncertain.

It is our current understanding that inserting implants or cement into the femoral canal has a significant impact on morbidity and mortality for patients having an arthroplasty procedure [24]. Most of the patients, in the study by Boddice et al., had open reduction and internal fixation as a surgical procedure for femoral periprosthetic fractures [5]. In the study by Singh et al., a higher mortality rate was observed in the revision arthroplasty group, but this was not statistically significant compared to the other groups [18]. These two studies considered that a revision arthroplasty procedure could potentially cause patients to have a higher mortality rate. In our study, more patients were subjected to revision surgery than fixation; revision surgery was carried out in 49 patients (55.68%) and 39 patients (44.32%) had fixation for their femoral periprosthetic fracture. The difference in mortality between the two groups in our study was not statistically significant.

No statistically significant difference, for the length of hospital and post-operative stay between the two groups, was found in our study; it was observed that the median length of hospital stay was greater for the patients who had surgery after 36 hours of diagnosis (13 days). The study by Boddapati et al. demonstrated that the length of hospital stay and post-operative stay were both higher for the group of patients who had delayed surgery after 24 hours [7]. Similarly, the studies by Boddice et al. and Singh et al., which were conducted in the UK, also found the patients to have a higher length of hospital stay with a delay in surgical intervention for femoral periprosthetic fractures [5, 18]. There are several patients in our study who stayed in the hospital, awaiting a rehabilitation placement despite being medically fit. This was a factor that we did not explore in our study. Length of hospital stay is often prolonged whilst waiting for rehabilitation and this could potentially contribute to our results not being statistically significant.

In our study, we observed that the post-operative complications were higher for the group of patients having their surgical intervention within 36 hours compared to the patients who had surgery after 36 hours; this was statistically significant with a p-value of 0.014. The study by Johnson-Lynn et al., which was carried out at two trusts in the UK, found that there was not a significant relationship between post-operative complications and a delay in surgical intervention [8]. In their study, Boddapati et al. observed a significantly higher complication rate in the group of patients who had delayed surgical intervention, particularly respiratory and urinary tract infections [7]. Furthermore, the post-operative complications were noted to be higher for the patients having surgery after 48 hours in the study by Singh et al., however, the results were not statistically significant [18].

The authors of this study feel that the results, regarding post-operative complications, not being consistent with the literature could be due to several factors. One of the factors could be the small sample size. There were only 15 patients (17.05%) who had surgery within 36 hours of diagnosis compared to the 73 patients (82.95%) having surgery after 36 hours. The two groups, perhaps, would have had a similar number of patients if the timing for surgical intervention of over 36 hours was selected in this study. Furthermore, our results also suggest that delaying surgical intervention, for medical optimisation and arranging appropriate specialist surgeon, leads to fewer post-operative complications.

The multiple regression demonstrated an association between CCI and one-year mortality. A correlation between age and one-year mortality was also observed in multiple regression analysis. These results are in keeping with the study by Finlayson et al. where the authors analysed the factors that predicted mortality after a femoral periprosthetic fracture [25]; they observed that there was a strong association between active neoplasia and one-year mortality. The patients, with cancer, have a higher CCI and we observed an association between CCI and one-year mortality in our study too. Finlayson et al. also mentioned in their study that patient mortality was observed in the older age group of patients [25]; we observed this finding in our study on multiple regression.

Having a small sample size is the main limitation of this study. The patient records were reviewed retrospectively; a prospective review of the patients would have improved the quality of evidence for this study. We also did not consider the delay in patients being discharged due to the waiting time for rehabilitation and this could be something that could be considered in future studies.

## Conclusions

Overall, the results of this study are consistent with the literature. The delay in surgical intervention for femoral periprosthetic fractures does not have a significant impact on patient mortality. We do not recommend any change of current practice. It is our view that careful planning for patients with femoral periprosthetic fractures leads to better patient outcomes, even if it results in delayed surgical intervention. A study with a larger sample size is required to analyse the impact of delayed surgical intervention on long-term mortality of over 10 years.
